# Immune-enhancing effects of anionic macromolecules extracted from *Codium fragile* coupled with arachidonic acid in RAW264.7 cells

**DOI:** 10.1371/journal.pone.0239422

**Published:** 2020-10-08

**Authors:** Chaiwat Monmai, Weerawan Rod-in, A-yeong Jang, Sang-min Lee, Seok-Kyu Jung, SangGuan You, Woo Jung Park

**Affiliations:** 1 Department of Marine Food Science and Technology, Gangneung-Wonju National University, Gangneung, Gangwon, Republic of Korea; 2 Department of Wellness-Bio Industry, Gangneung-Wonju National University, Gangneung, Gangwon, Republic of Korea; 3 Department of Marine Biotechnology, Gangneung-Wonju National University, Gangneung, Gangwon, Republic of Korea; 4 Department of Horticulture, Daegu Catholic University, Gyeongsan, Gyeongbuk, Republic of Korea; University of Missouri Columbia, UNITED STATES

## Abstract

Arachidonic acid (ARA) is an integral constituent of the biological cell membrane, conferring it with fluidity and flexibility, which are necessary for the function of all cells, especially nervous system, skeletal muscle, and immune system. *Codium* species biosynthesize sulfated polysaccharides with very distinct structural features. Some of them have different biological activities with great potential in pharmaceutical applications. In this study, anionic macromolecules extracted from *Codium fragile* were investigated for their cooperative immune-enhancing activities with ARA. The cooperation between ARA and Codium resulted in increased, dose-dependent nitric oxide production and iNOS gene expression. In addition, co-treatment of ARA and Codium effectively increased pro-inflammatory cytokines (IL-1β, IL-6, and TNF-α), compared with Codium alone. We also demonstrated that the expression of COX-2 mRNA was also increased, which is responsible for the production of inflammatory mediator prostaglandins and their metabolites. Compared to the Codium group, the co-treatment of Codium with ARA enhanced the phosphorylation of nuclear factor-κB p-65, p38, and extracellular signal-related kinase 1/2, indicating that this combination stimulated immune response through nuclear factor-κB and mitogen-activated protein kinase pathways. These results indicated that the coordination of arachidonic acid with polysaccharide extracted from seaweed may be a potential source of immunomodulatory molecules.

## 1. Introduction

The human immune system was developed to protect the host from infection by foreign materials and pathogens [1]. It has a highly complex structure to eliminate invaders, as well as to help repair infected or damaged sites to restore homeostasis [2]. Macrophages play a role in one of the important parts of the immune system [3], and perform several biological activities such as host defense, inflammation control, and remodeling of tissue [4]. Macrophages exhibit different phenotypes at the different stages of the inflammatory response [5]. Macrophages have at least two different polarizations, the classical (M1) and alternative (M2) [6, 7]. M1 and M2 macrophages can provide their biological activities by secreting different cytokines and effector molecules [8]. The activation of M1 macrophages is associated with regulation of nitric oxide synthase (iNOS) via production of nitric oxide (NO), which is important for removal of infection and cytokine secretion for antigen defense, including anti-bacterial, anti-viral, and anti-tumor functions [9–13]. The activation of macrophages is thought to help resist infection [14]. The activation of M2 macrophage relate to the natural inflammation resolution. Therefore, M2 macrophages are usually mentioned as having repairing or anti-inflammatory functions [15].

Fatty acids are classified as saturated, monounsaturated, or polyunsaturated fatty acids (PUFA) by their degree of saturation. PUFAs such as dihomo-gamma-linolenic acid (DGLA), arachidonic acid (ARA, 20:4n-6) and eicosapentaenoic acid (EPA, 20:5n-3) are the precursors of eicosanoids which include prostaglandins (PG), thromboxanes (TX), lipoxins (LX), and leukotrienes (LT) [16, 17]. These compounds are associated with the host defense system against infections including resistance to stress stimuli, the immune response, and inflammatory processes [17]. Normally, ARA plays an important role in inflammatory processes and stimulates the production of pro-inflammatory cytokines [18], whereas EPA plays a key role in the anti-inflammatory process [19–21]. ARA is generally the preferred substrate of lipoxygenases for LT synthesis and products derived from these enzymes produce strong immune stimulatory effects [22, 23]. Arachidonic acid metabolism also leads to the synthesis of prostaglandins (PGs), which are one of the lipid mediators [22]. PGs are involved in the immune response via gene regulation of cytokine signaling such as TNF-α and IL-1β [24].

*Codium fragile* is a green seaweed belonging to the family *Codiaceae*, which is spread along the shores of East Asia, Oceania, and North Europe [25]. It has been shown that the extracted compounds of *C*. *fragile* show several biological functions, such as anti-coagulant [26], anti-viral [27], anti-inflammatory [28], and immunomodulatory activities [29]. Very recently, our group reported that anionic molecules extracted from *C*. *fragile* acted on cyclophosphamide immune-suppressed mice. The current study investigated if the anionic macromolecules (Codium) extracted from *C*. *fragile* enhanced immunity when co-treated with ARA on RAW264.7 cells, compared with treatment without ARA.

## 2. Materials and methods

### 2.1 Chemicals

Arachidonic acid was purchased from Nu-Check Prep, Inc. (USA). Griess reagent was purchased from Sigma-Aldrich (USA). The EZ-Cytox Cell Viability Assay Kit was purchased from Daeil Labservice (Seoul, South Korea). The Tri reagent® was purchased from Molecular Research Center, Inc (Ohio, USA). A high capacity cDNA reverse transcription kit was purchased from Applied Biosystems (California, USA). SYBR® Premix Ex Taq™ II was purchased from Takara Bio Inc. (Kusatsu, Japan). Radioimmunoprecipitation assay (RIPA) buffer was purchased from Tech & Innovation (Hebei, China). The Pierce™ BCA Protein Assay Kit and Pierce® ECL Plus Western Blotting Substrate were purchased from ThermoScientific (Waltham, USA). Specific antibodies for p-NF-κB p65, p-p38, p-ERK1/2 and p-JNK were purchased from Cell Signaling Technology (Danvers, USA) and α-Tubulin was purchased from Abcam (Cambridge, United Kingdom).

### 2.2 Isolation of crude anionic macromolecules

Anionic macromolecules from *C*. *fragile* (Codium) were isolated as described previously [30]. Briefly, Codium was extracted from the milled sample of *C*. *fragile* using 80% EtOH and then dried at room temperature in a fume hood. The dried biomass was extracted with distilled water at 65°C with stirring for 2 h. The supernatant was collected and then concentrated by evaporation under reduced pressure at 60°C to approximately 200 mL. EtOH (99%) was added into the supernatant to obtain a final EtOH concentration of 70% and then kept at 4°C overnight. The precipitate was obtained by filtration of the solution. The precipitate was washed with EtOH (99%), followed by acetone, and then dried at room temperature in a fume hood. The recovered precipitate was re-dissolved in distilled water, and then the removal of free-proteins in the precipitate was carried out using the Sevag method to confirm that the proteins included in the precipitate were covalently bound [31].

### 2.3 Macrophage proliferation and nitric oxide production

Murine macrophages (1×10^5^ cells), RAW264.7 cells (Korean Cell Line Bank), in RPMI-1640 medium (supplemented with 10% FBS and 1% penicillin/streptomycin) were placed in a 96-well plate. The plate was incubated for 24 h at 37°C in an atmosphere of 5% CO_2_. The different concentrations of Codium (0, 0.5, 1, 2, and 4 μg/mL) augmented with 0.5 μM of ARA were added to the macrophages and the plate was incubated for another 24 h. Cells grown in the presence of 1 μg/mL LPS were used as a positive control. All experiments were performed in triplicate. Nitric oxide (NO) production was determined by measuring the quantity of nitrite in the cell culture medium using the Griess reagent [32] and compared with standard curve. The EZ-Cytox Cell Viability Assay kit was used to analyze cellular proliferation [33]. The cellular proliferation ratio (%) was calculated based on the following formula:

$$\text{Cell}\;\text{proliferation}\;\text{ratio}\;(\%)=\frac{{\text{A}}_{450}\text{of}\;\text{the}\;\text{test}\;\text{group}}{{\text{A}}_{450}\text{of}\;\text{the}\;\text{control}\;\text{group}}\times 100$$

Where; A_450_ = absorbance at 450 nm.

### 2.4 RNA extraction and cDNA synthesis

Total RNA was extracted from treated cells using the Tri reagent®. RNA was precipitated using 100% isopropanol and washed with 75% EtOH. The RNA concentration was analyzed using the nanophotometer (Implen, Germany). First strand cDNA was synthesized using the High Capacity cDNA Reverse Transcription kit according to the manufacturer’s instructions.

### 2.5 Quantitative Real-Time PCR

Quantification of RAW264.7 immune gene expression was performed using SYBR® Premix Ex Taq™ II. The reaction mixture consisted of 0.4 μM of each specific primer pair (Table 1) and 0.1 ng of cDNA template. Quantitative Real-Time PCR was performed and analyzed with the QuantStudio 3 Flex real‐time PCR system (Applied Biosystems, Foster City, CA) using a relative standard curve method compared with β-actin as an internal control of immune gene expression.

**Table 1:** Oligonucleotide primers used in real-time PCR for evaluating immune gene expression.

Gene	Accession No.	Oligonucleotide Sequence (5ʹ - 3ʹ)
iNOS	BC062378.1	Forward: TTCCAGAATCCCTGGACAAG
		Reverse: TGGTCAAACTCTTGGGGTTC
IL-1β	NM_008361.4	Forward: GGGCCTCAAAGGAAAGAATC
		Reverse: TACCAGTTGGGGAACTCTGC
IL-6	NM_031168.2	Forward: AGTTGCCTTCTTGGGACTGA
		Reverse: CAGAATTGCCATTGCACAAC
TNF-α	D84199.2	Forward: ATGAGCACAGAAAGCATGATC
		Reverse: TACAGGCTTGTCACTCGAATT
IFN-γ	NM_008337.3	Forward: CTCAAGTGGCATAGATGT
		Reverse: GAGATAATCTGGCTCTGCAGGATT
COX-2	NM_011198.4	Forward: AGAAGGAAATGGCTGCAGAA
		Reverse: GCTCGGCTTCCAGTATTGAG
TLR-4	NM_021297.3	Forward: CGCTCTGGCATCATCTTCAT
		Reverse: GTTGCCGTTTCTTGTTCTTCC
GPR120	AY288424.1	Forward: GATGACAATGAGCGGCAGCG
		Reverse: GATTTCTCCTATGCGGTTGGG
β-actin	NM_007393.5	Forward: CCACAGCTGAGAGGGAAATC
		Reverse: AAGGAAGGCTGGAAAAGAGC

### 2.6 Western blot assay

RAW264.7 cells were harvested with RIPA buffer. The protein concentration was measured using the Pierce™ BCA Protein Assay kit and proteins were separated by sodium dodecyl sulfate-polyacrylamide gel electrophoresis (SDS-PAGE). The proteins were transferred to a polyvinylidene fluoride (PVDF) membrane, and a western blot assay was performed, as described by Narayanan et al. [34]. Specific antibodies were used for p-NF-κB p65, p-p38, p-ERK1/2, p-JNK, and α-Tubulin. Signals were recognized using the Pierce® ECL Plus Western Blotting Substrate. The blot was quantitatively analyzed using the ChemiDoc XRS+ imaging system (Bio-Rad) and ImageLab software (version 4.1, Bio-Rad).

### 2.7 Statistics

Statistics Software, Statistix 8.1, was used for statistical analysis and values were evaluated by one-way analysis of variance, followed by post-hoc Duncan’s multiple range tests. The differences between the two groups were compared using *t*-tests (*p* < 0.05).

## 3. Results

### 3.1 Codium coupled with ARA enhanced NO production in RAW264.7 cells without any cytotoxicity

**Fig 1:**
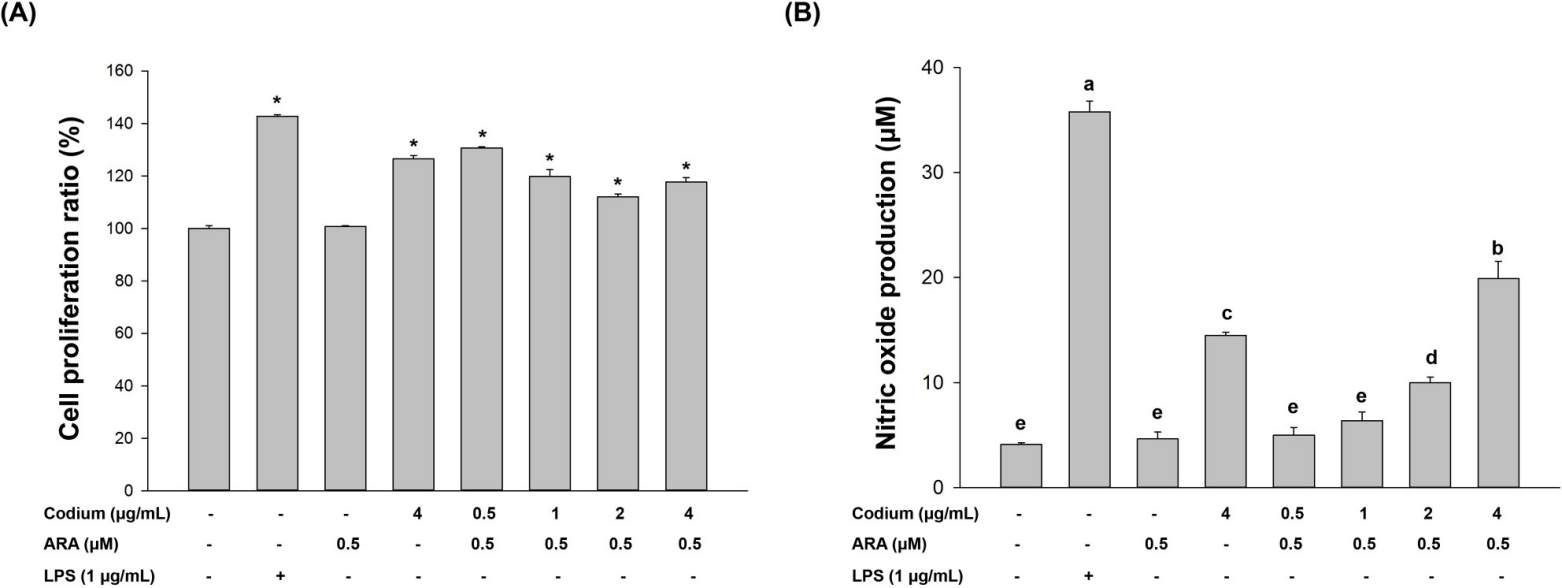
Co-operative effect of Codium and ARA in RAW264.7 cells. (A) Effect on macrophage proliferation (*, *p* < 0.05 compared to the RPMI group) and (B) effect on nitric oxide production (The letters a,b,c,d,e indicate a significant difference (*p* < 0.05) between the amount of NO production).

The Codium composition was previously reported from our research group [30]. The main consist of Codium is carbohydrates (54.6%) and consisted of protein (15.7%), sulfates (13.0%), and uronic acid (1.4%). The major sugar of Codium was measured using monosaccharide composition analysis and the highest was galactose (59.5%). Fig 1A showed no obvious cytotoxicity of Codium (up to 4 μg/mL) in RAW264.7 cells irrespective of presence of ARA. As shown in Fig 1B, treatment of Codium coupled with ARA significantly increased NO production, depending on the Codium concentration. Moreover, the treatment of Codium with 0.5 μM ARA also increased NO production, compared with treatment of Codium or ARA alone.

### 3.2 Codium coupled with ARA enhanced immune associated gene expression in RAW264.7 cells

**Fig 2:**
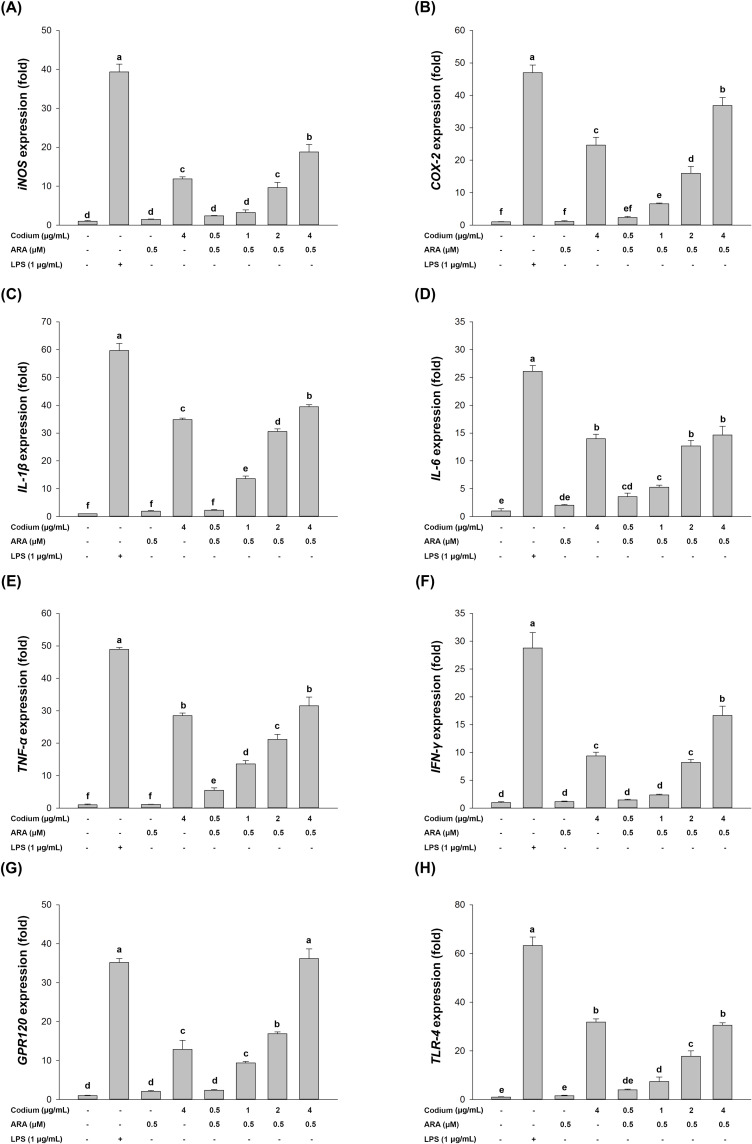
Relative quantitation of immune gene expression (fold-change). Expression of (A) iNOS, (B) IL-1β, (C) IL-6, (D) TNF-α, (E) IFN-γ, (F) COX-2, (G) GPR120, and (H) TLR-4. The letters a,b,c,d,e,f indicate a significant difference (*p* < 0.05) between the treatments.

Treatment of 4 μg/mL Codium to RAW264.7 cells resulted in enhanced production of immune-associated genes (Fig 2). Furthermore, these gene expression results indicated that incubation of RAW264.7 cells with Codium in addition to 0.5 μM ARA, led to an increase in expression of most immune-associated genes, depending on the concentration of Codium. Finally, co-treatment of Codium and ARA led to a higher target gene expression than treatment with Codium alone. Most of all, GPR120 which is a receptor for polyunsaturated fatty acids, was expressed in higher amounts than with Codium alone.

### 3.3 Codium coupled with ARA enhanced immunity through the MAPK and NF-κB signaling pathways in RAW264.7 cells

**Fig 3:**
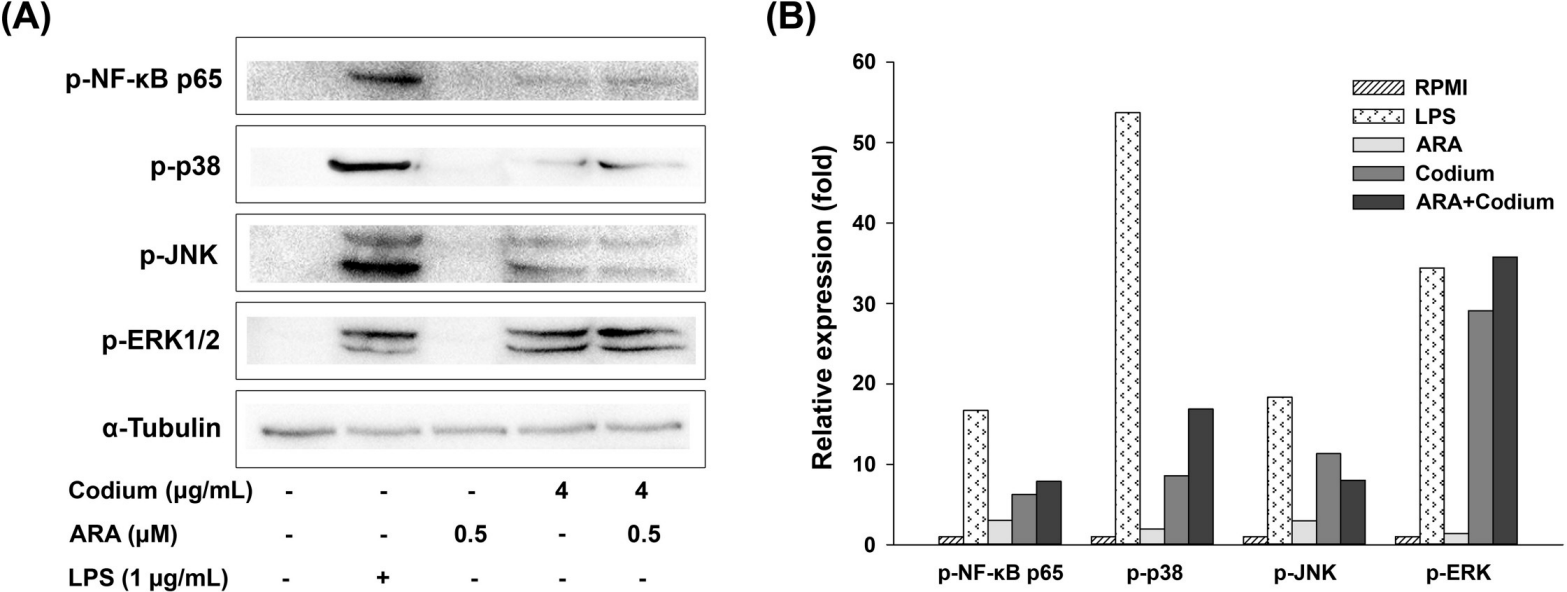
Co-operative effect of Codium and ARA on proteins associated with NF-κB and MAPK pathways. (A) Expression of proteins by western blot and (B) relative band intensity of proteins.

The production of NF-κB and MAPK associated proteins were analyzed through western blotting to determine the contribution of Codium and ARA on the immune-enhancement in RAW264.7 cells. Treatment of Codium stimulated the phosphorylation of NF-κB p-65, compared to normal cells (Fig 3). Codium also increased the phosphorylation levels of ERK1/2 and p38 of the MAPK pathway. Interestingly, phosphorylation of NF-κB p-65, ERK1/2, and p38 were increased in RAW264.7 cells with the treatment of Codium coupled with ARA.

## 4. Discussion

Macrophages play important roles in host defense, inflammation control [4], combating infection, and removing tumor cells in hosts [35]. Increased production of nitric oxide (NO) by macrophages protects host cells from foreign infection [14]. The nitrite-derived NO production also activates immune cells for the production of pro-inflammatory cytokines, such as TNF-α and IL-1β [36]. It was confirmed that the mixture of Codium (up to 4 μg/mL) and 0.5 μM ARA did not affect the growth of RAW264.7 cells in terms of cell proliferation, revealing that there was no synergistic or individual cytotoxicity among these two supplements against RAW264.7 cell division (Fig 1). Codium is known to stimulate one of the important immune regulatory biomarkers, NO production, in macrophage cells [30], however the action of Codium coupled with ARA has not been reported. The current study showed that the combination of Codium and ARA significantly enhanced the production of NO in a dose-dependent manner and also resulted in higher NO production than with treatment of Codium alone.

Cytokine expression is another critical factor for the investigation of immunity, inflammation and hematopoiesis [37]. Activated macrophages secrete cytokines such as TNF-α, IL-1β and IL-6 [37–39] to defend against intracellular pathogens [40]. Normally, n-6 fatty acid plays an important role in pro-inflammatory processes by stimulating the production of pro-inflammatory cytokines (TNF-α, IL-1β, and IL-6) [16, 17, 41]. The current results showed that most of the immune-associated genes were significantly enhanced with the supplementation of ARA, compared to treatment of Codium alone (Fig 3), suggesting that ARA helped Codium to increase the expression of immune-associated genes in RAW264.7 cells. Interestingly, the expression of TLR-4, an important polysaccharide receptor [42, 43], did not show significant difference between the treatment of Codium alone or in combination with ARA, whereas the expression of GPR120, the receptor for polyunsaturated fatty acids, was significantly increased [21, 44, 45]. These results indicated that the increased expression of immune-associated genes was affected by the incorporation of ARA.

*Codium fragile*, one of the popular algae, has been employed for oriental therapy medicine to treat diseases such as enterobiosis, dropsy, and dysuria [25]. Moreover, this algae was demonstrated to have immune-regulatory activities such as anti-inflammatory, and immune-stimulatory activities [20, 33]. In addition, ARA is capable of activating the GPR120 receptor, which is related to calcium mobilization and ERK [46] and p38 stimulation [47], thus leading to health-promoting effects [21, 48, 49]. To regulate the immune response in macrophage cells, NF-κB, and MAPK pathways are coordinated via the phosphorylation of the associated proteins in these pathways [50]. That is, NF-κB coordinates the expression of pro-inflammatory mediators and pro-inflammatory cytokines [51], and MAPK-related molecules play critical roles in regulating cell growth and differentiation, and controlling cellular responses to cytokines [52]. Therefore, our results demonstrated that the synergistic effect between Codium and ARA led to activation of NF-κB, p-65, and MAPK such as ERK1/2 and p38, and thus promoted the immune response in RAW 264.7 cells.

## 5. Conclusions

In conclusion, it was demonstrated that the coordination of Codium and ARA synergistically enhanced the expression and production of biomarkers such as production of NO and the expression of immune-associated genes, such as iNOS, IL-1β, TNF-α, IFN-γ, and COX-2 in RAW264.7 cells. Furthermore, these increases were triggered by activation of NF-κB p-65 and MAPK, including ERK1/2 and p38, thus regulating the immune responses for immune-enhancement. These results suggested that incorporating ARA into Codium would contribute to an improved immune response in RAW264.7 cells.

## Supporting information

S1 File (PDF)
