# Supplementary figures and images for: Immune-enhancing effects of anionic macromolecules extracted from Codium fragile coupled with arachidonic acid in RAW264.7 cells
Source: PLoS One. 2020 Oct 8;15(10):e0239422. doi: 10.1371/journal.pone.0239422 (PMC7544070; doi:10.1371/journal.pone.0239422)

**Figure 3**

**p-ERK and  $\alpha$ -Tubulin**

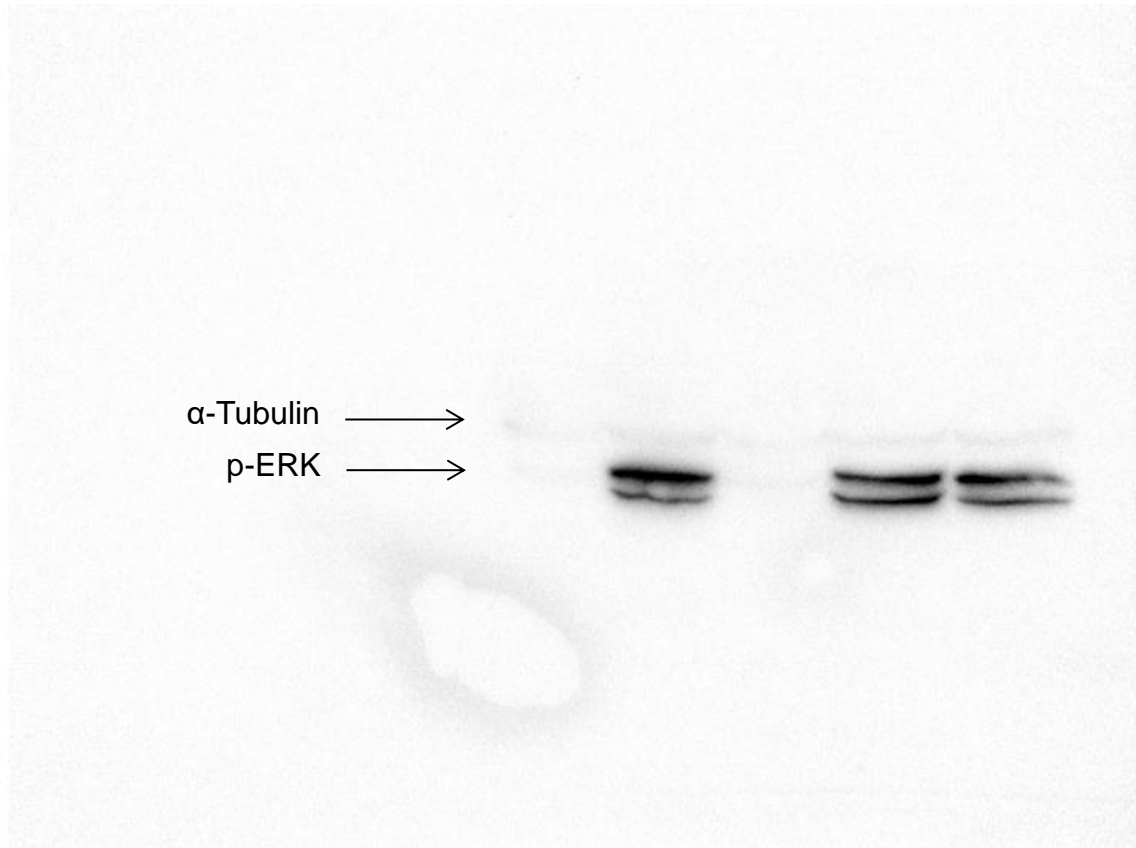

**p-p65 and p-p38**

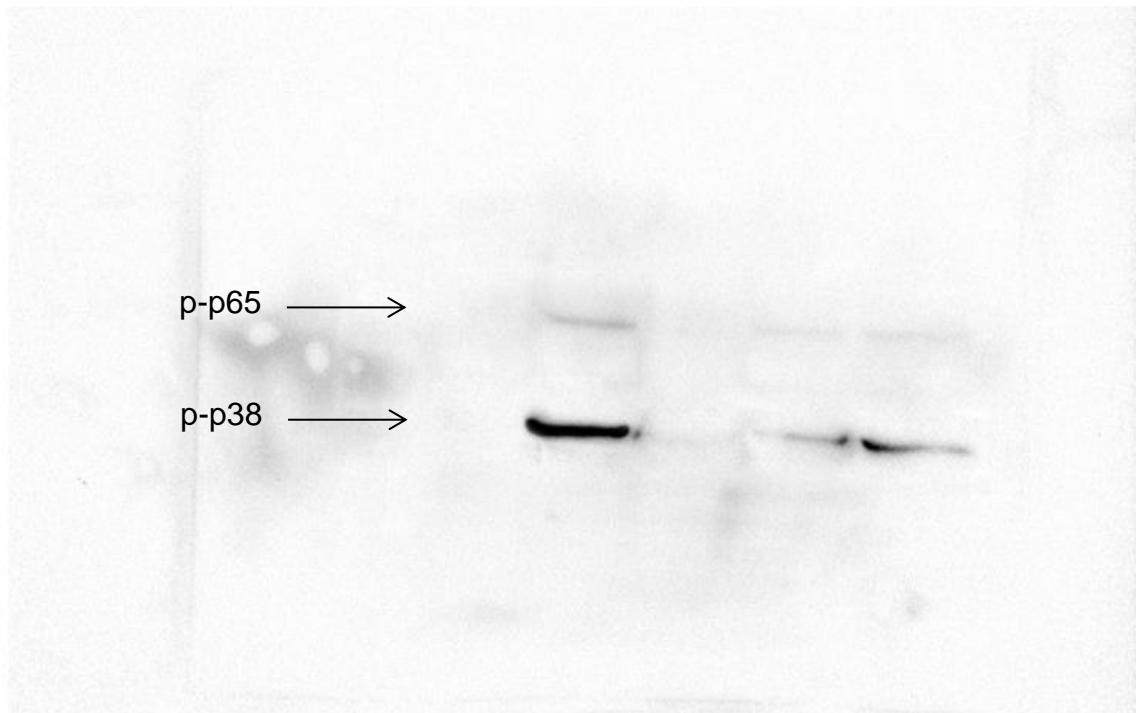

p-JNK

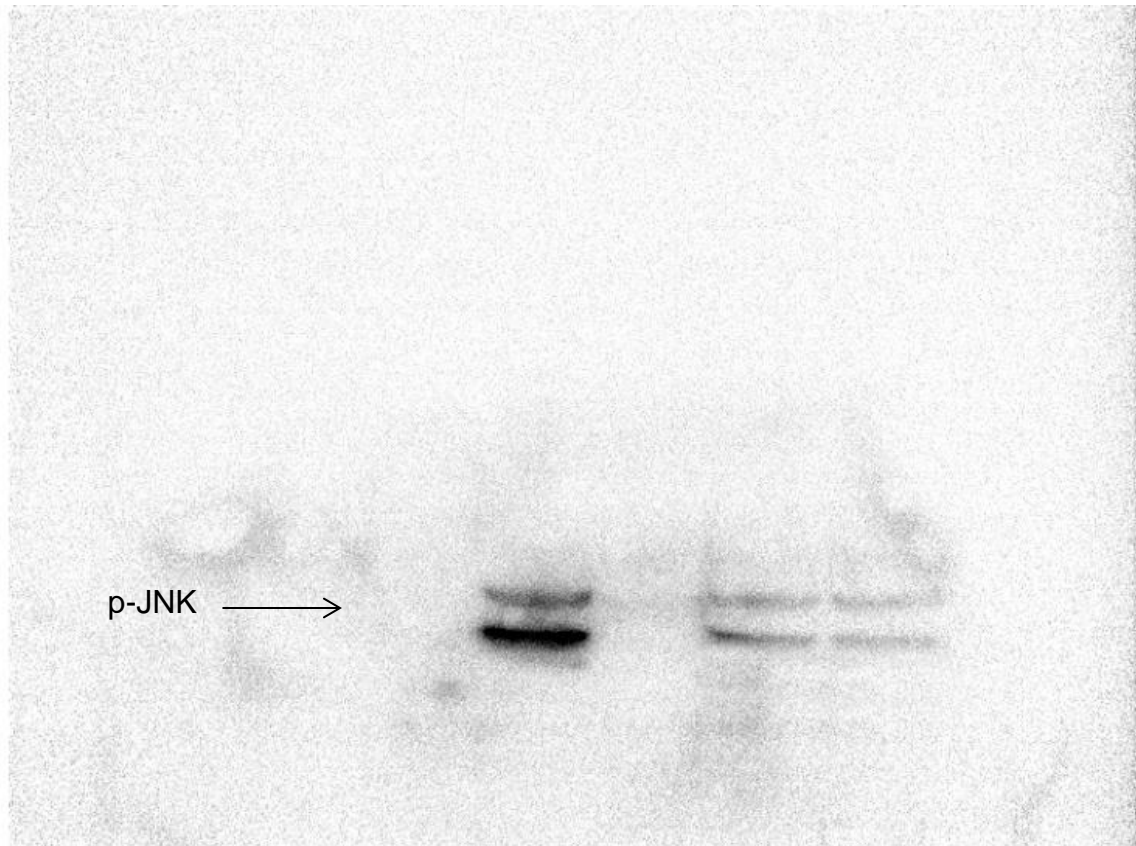

Supplement: S1 File — (PDF) [file pone.0239422.s001.pdf]
